# Evolutionary changes of noncoding elements associated with transition of sexual mode in *Caenorhabditis* nematodes

**DOI:** 10.1126/sciadv.adn9913

**Published:** 2024-09-13

**Authors:** Katsunori Tamagawa, Mehmet Dayi, Simo Sun, Rikako Hata, Taisei Kikuchi, Nami Haruta, Asako Sugimoto, Takashi Makino

**Affiliations:** ^1^ Graduate School of Life Sciences, Tohoku University, Aoba-ku, Sendai, Japan.; ^2^ Forestry Vocational School, Duzce University, 81620 Duzce, Türkiye.; ^3^ Graduate School of Frontier Sciences, The University of Tokyo, Kashiwanoha, Kashiwa City, Japan.; ^4^ Department of Biology, Faculty of Science, Tohoku University, Aoba-ku, Sendai, Japan.

## Abstract

The transition of the sexual mode occurs widely in animal evolution. In *Caenorhabditis* nematodes, androdioecy, a sexual polymorphism composed of males and hermaphrodites having the ability to self-fertilize, has evolved independently multiple times. While the modification of noncoding regulatory elements likely contributed to the evolution of hermaphroditism, little is known about these changes. Here, we conducted a genome-wide analysis of conserved noncoding elements (CNEs) focusing on the evolution of hermaphroditism in *Caenorhabditis* nematodes. We found that, in androdioecious nematodes, mutations rapidly accumulated in CNEs’ neighboring genes associated with sexual traits. Expression analysis indicate that the identified CNEs are involved in spermatogenesis in hermaphrodites and associated with the transition of gene expression from dioecious to androdioecious nematodes. Last, genome editing of a CNE neighboring *laf-1* resulted in a change in its expression in the gonadal region undergoing spermatogenesis. Our bioinformatic and experimental analyses highlight the importance of CNEs in gene regulation associated with the development of hermaphrodites.

## Teaser

Conserved noncoding elements in the genome play a vital role in gene regulation associated with the evolution of hermaphroditism.

## INTRODUCTION

Transitions of sexual reproduction mode occur in various lineages of organisms and are one of the most intriguing puzzles in ecology, evolutionary biology, and genomics (*1*). In animals, sexual reproduction between male and female predominates, but transitions between dioecy and hermaphroditism occur widely in animals (*2*, *3*). The transition of reproductive modes in evolution is accompanied by a wide range of biological changes, including habitat, behavior, and morphology.

**Fig. 1.**
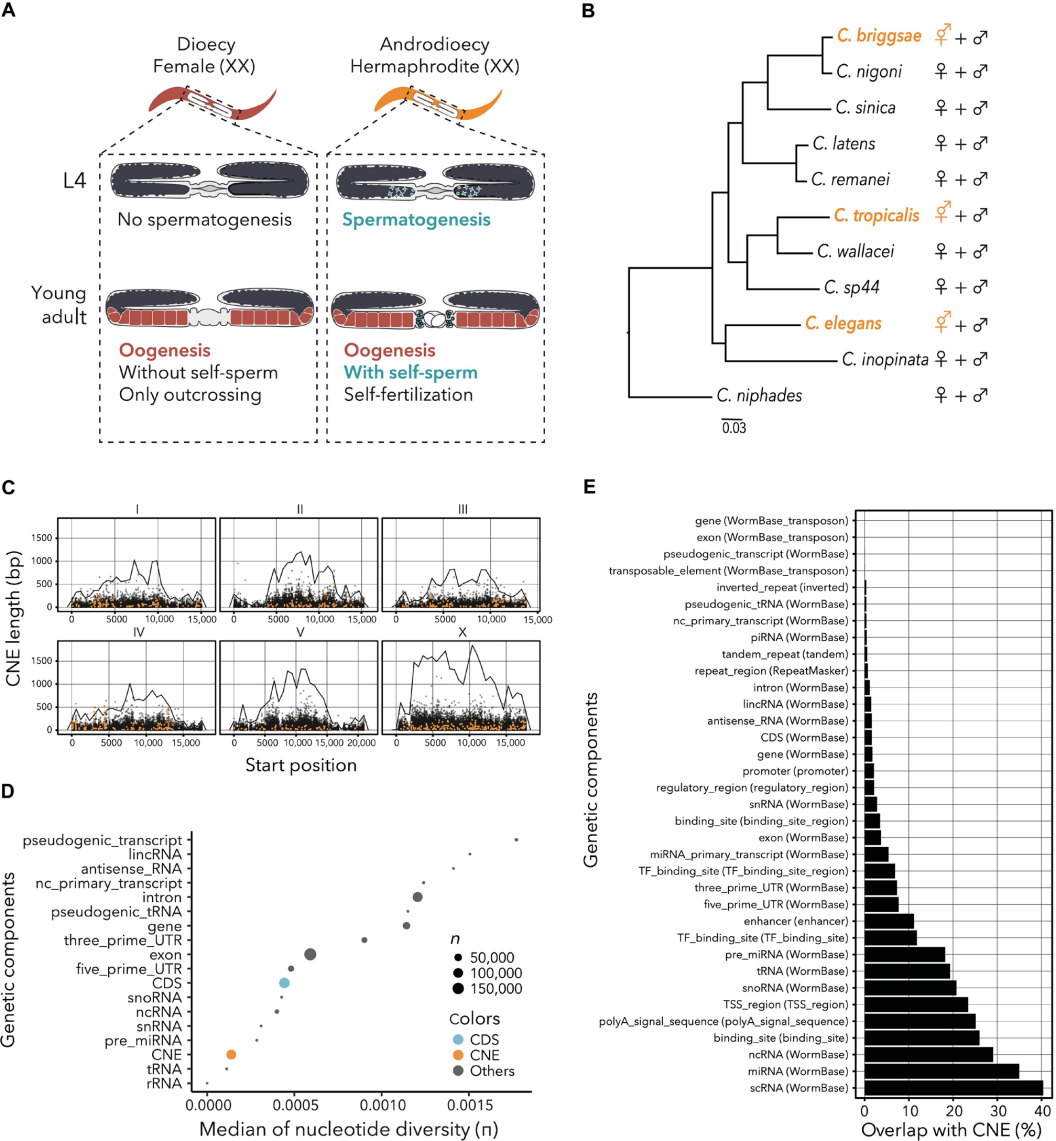
Evolution of hermaphroditism in *Caenorhabditis* nematodes and the position and sequence characteristics of CNEs. (**A**) Schematic illustration of germ cell development of the female of dioecy (left) and hermaphrodite of androdioecy (right). (**B**) Phylogenetic tree of *Caenorhabditis* nematodes constructed using single-copy orthologs by maximum likelihood estimation. The branch length corresponds to sequence substitution and the species indicated in orange represent androdioecious nematodes. (**C**) Distribution and length of CNEs in each chromosome of *C. elegans*. Each dot represents the start position and length of CNEs, and the orange color represents CNEs under accelerated evolution. The line shows CNE frequency across the chromosomes. (**D**) The nucleotide diversity of the CNEs and each genetic component in the wild *C. elegans* strains deposited in CeNDR. The size of each point represents the number of sequences used to calculate the nucleotide diversity. To reduce the effect of sequences that were too short, median values were calculated using only sequences >50 bp. Moreover, genetic components containing more than 10 are shown. The orange and blue points represent the median of nucleotide diversity of CNEs and coding sequences (CDSs), respectively. (**E**) The overlap of the CNEs and each genetic component annotated in the general feature format (gff) file of *C. elegans* in WormBase. The horizontal axis represents the percentage of each sequence overlapping with CNEs in the whole genome. Several parts of CDSs overlapped with the CNEs in *C. elegans* genome because the CNEs were detected using *C. niphades* as a reference. piRNA, PIWI-interacting RNA; lincRNA, long intervening/intergenic noncoding RNA; snRNA, small nuclear RNA; snoRNA, small nucleolar RNA; scRNA, small cytoplasmic RNA.

Androdioecy (sexual polymorphism of males and hermaphrodites) of *Caenorhabditis* nematodes including the model nematode *C. elegans* can be an excellent model to study the evolutionary transition of sexual mode because the transition has occurred independently multiple times within the genus, namely, in the branches leading to *C. elegans*, *C. briggsae*, and *C. tropicalis* (*4*). *Caenorhabditis* nematodes use genetic sex determination, where XX is female and XO is male. In the hermaphroditic species, XX individuals produce sperm temporarily at the late larval stage and oocytes at the adult stage, which enables self-fertilization (*5*, *6*) (Fig. 1A). Recently, a new dioecious species, *Caenorhabditis inopinata*, was discovered in Japan as a sibling species of *C. elegans* (*7*). The availability of detailed genomic information and various genetic techniques of the model nematode *C. elegans* and the accumulation of genome sequence data of its dioecious and androdioecious relative species make the genus *Caenorhabditis* a powerful model for understanding the evolution of sexual mode.

Regulatory changes in sex-determination pathways are important for the transition of sexual mode. The sex-determination pathways of somatic/germline cells of *C. elegans* have been investigated in detail, and several genes are known to control spermatogenesis in hermaphrodites (*8*–*10*). The alteration of spatiotemporal regulation of transcription and translation appears to contribute to the evolution of hermaphroditism. However, information is limited regarding the association between evolutionary changes in regulatory elements in the genome and the transition of sexual mode in nematodes.

Non–protein-coding genomic sequences conserved during evolution often have regulatory functions for proper organismal development and maintenance (*11*–*13*). These conserved noncoding elements (CNEs) are likely to be under evolutionary constraints because they overlap with functional sequence units (*11*–*14*). Similar to amino acid changes in protein-coding genes, nucleotide changes in CNEs also play important roles in phenotypic evolution. Recently, several studies suggested that lineage-specific rapid mutation accumulation (accelerated evolution) in CNEs contributes to large phenotypic changes in vertebrates (*15*–*17*). Although evolutionary changes in CNEs of invertebrates have not been extensively studied, mutation accumulations in CNEs might also contribute to phenotypic evolution in invertebrates (*18*, *19*).

Here, we examined the association between CNEs and the evolution of hermaphroditism using whole-genome sequences of *Caenorhabditis* nematodes. To further assess the influence of evolutionary changes in CNEs, we performed transcriptomic analysis of androdioecious *C. elegans* and its closest dioecious relative, *C. inopinata*. Genome editing of an identified CNE was used to assess its effect on gene expression of neighboring genes. Our analysis provides insights into the effect of evolutionary changes in regulatory elements on the transition of sexual mode.

## RESULTS

### Genome alignment and detection of CNE in *Caenorhabditis* nematodes

In *Caenorhabditis* nematodes, the ability of self-fertilization in androdioecious species has evolved convergently (Fig. 1, A and B). The phylogenetic tree of 11 species estimated here consisted of well-known phylogenetic relationships, and the bootstrapping values were adequately high overall (Fig. 1B). In this analysis, 133,541 CNEs were obtained upon aligning the *Caenorhabditis* genome with that of *C. niphades*, the outgroup with a high-quality genome sequence, as a reference to avoid the bias in CNE detection in subject species. After filtering for CNE length and duplication, 73 to 85% remained in each species (table S1). Consistent with gene density in the *C. elegans* genome, CNEs were most abundant in the centers of the chromosomes (Fig. 1C). The number and percentage of base pairs of CNEs in the noncoding region were highest in the X chromosome in the *C. elegans* genome, which was more than twice as high as in any other chromosome (table S2). To examine the degree of the CNE conservation among different strains of *C. elegans* in the wild population, nucleotide diversity was calculated for each CNE as well as for other genomic elements [exon, intron, untranslated region (UTR), noncoding RNA, etc.]. The nucleotide diversity of the CNEs tended to be low among *C. elegans* in the wild population compared with other genomic elements including protein-coding sequences (Fig. 1D). Small noncoding RNAs and regulatory elements abundantly overlapped with the CNEs whereas long noncoding RNAs did not (Fig. 1E). Probably because of the difficulty of aligning the sequences, the repetitive elements overlapped only slightly with the CNEs (Fig. 1E).

### Accelerated evolution of CNE in three hermaphrodite nematodes

We detected 711 CNEs in three hermaphrodite species (ha-CNEs) as having undergone accelerated evolution when all three branches were targeted in parallel [false discovery rate (FDR) < 0.05]. In this approach, the CNEs exhibiting weak or no accelerated evolution in several branches may have been included, leading to the frequent detection of accelerated evolution shared by only two branches. The ha-CNEs tended to overlap with enhancer regions presumed in *C. elegans* (*20*, *21*) (table S3, one-sided Fisher’s exact test, FDR = 0.07356). Although promoter and UTR regions are associated with gene regulation, we found no significant overlap of ha-CNEs with either promoter, 5**′** or 3**′** UTR regions (table S3, one-sided Fisher’s exact test FDR = 0.9196 for promoter, FDR = 0.9196 for 5**′** UTR, and FDR = 0.9196 for 3**′** UTR). To further investigate the functional association of ha-CNEs with the evolution of hermaphroditism, 613 protein-coding genes were extracted as the closest neighbors of the ha-CNEs (table S4). The nucleotide diversity of these genes did not vary notably compared to that of other genes (fig. S2). These genes were enriched in functions of sexual dimorphism and male development according to gene set enrichment analysis (GSEA) of phenotype association implemented in WormBase (table S5). The GSEA of tissue association showed that genes related to various neuron-associated terms were enriched in the genes neighboring ha-CNEs (table S5). In gene ontology, some terms related to neuronal development were enriched (table S5).

### Independent accelerated evolution in each hermaphrodite species

The independent accelerated evolution of CNEs in each species might contribute to hermaphrodite evolution. We focused on the accelerated evolution of each hermaphrodite species, and 2525, 898, and 1385 CNEs were detected in *C. elegans*, *C. briggsae*, and *C. tropicalis*, respectively (FDR < 0.05). In this assessment, no CNE under accelerated evolution was detected in all three hermaphrodite species. However, we found several CNEs under accelerated evolution in two hermaphrodite species (fig. S3). The number of overlapping CNEs was lower in the intersection with *C. briggsae* than between *C. elegans* and *C. tropicalis* (fig. S3). In several cases, we found that different CNEs under accelerated evolution in each species were independently identified to neighbor the same orthologs (23 orthogroups). These orthologs were significantly enriched in genes associated with sexually dimorphic behavior and transcription factor binding (tables S6 and S7). Four of 23 orthologs, i.e., *egl-19* (*22*), *egl-27* (*23*), *madd-2* (*24*, *25*), and *lsy-22* (*26*), are associated with phenotypes such as egg laying, vulval development, and male fertility in *C. elegans* (*27*). Because the evolution of hermaphrodites has been accompanied by many changes, not only in the germ line but also in somatic cells, these genes could be associated with hermaphrodite evolution.

### Accelerated evolution neighboring sex determination pathway genes

**Fig. 2.**
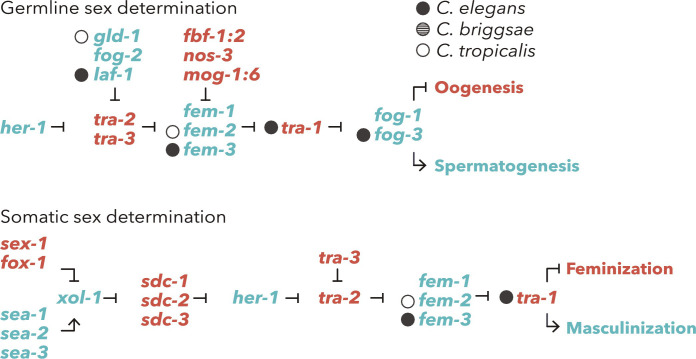
Sex-determination pathways in *C. elegans* and accelerated evolution of neighbor CNEs. The circles next to gene names correspond to the species with detected accelerated evolution of CNEs. Notably, one of the germline sex determination genes, *fog-2*, is known to be uniquely found in *C. elegans*.

To further inspect the association of CNEs and hermaphroditism, we analyzed the CNEs neighboring somatic and germline sex-determination genes previously identified in *C. elegans* (Fig. 2). No gene neighboring the CNEs with accelerated evolution was found in all three species, and we could not find gene-level convergent changes among species. In *C. briggsae*, no CNE with accelerated evolution was detected in neighboring genes associated with sex determination in *C. elegans*. We did not detect the sex-determination genes previously identified in *C. briggsae* neighboring the CNEs with accelerated evolution (*trr-1*, *cul-2*, and *puf-8*) (*28*). However, the evolution of the CNEs neighboring *laf-1*, *tra-1*, *fem-3*, and *fog-3* in *C. elegans* and the evolution of CNEs neighboring *fem-2* and *gld-1* in *C. tropicalis* were accelerated (Fig. 2). For *C. elegans*, the CNEs neighboring *laf-1*, *tra-1*, *fem-3*, and *fog-3* are located in the 3′ UTR, intergenic, intergenic, and upstream regions, respectively. For *C. tropicalis*, both CNEs neighboring *fem-2* and *gld-1* are located in the 3′ UTR. The accelerated CNEs neighboring *fem-2* and *fem-3* were detected in each species, and those gene products are assumed to control *tra-1* transcription by interacting with each other in both somatic and germline sex determination (*29*). In addition, we found accelerated CNEs neighboring the germ line–specific sex-determination genes, *laf-1* in *C. elegans* and *gld-1* in *C. tropicalis* (Fig. 2). Both gene products negatively regulate *tra-2* activity and induce temporary spermatogenesis in hermaphrodites (*9*). These results suggest that evolutionary convergence in different genes of the same pathway may have been associated with the evolution of hermaphroditism in at least two species.

### The effect of accelerated evolution of CNE on expression of neighboring gene

**Fig. 3.**
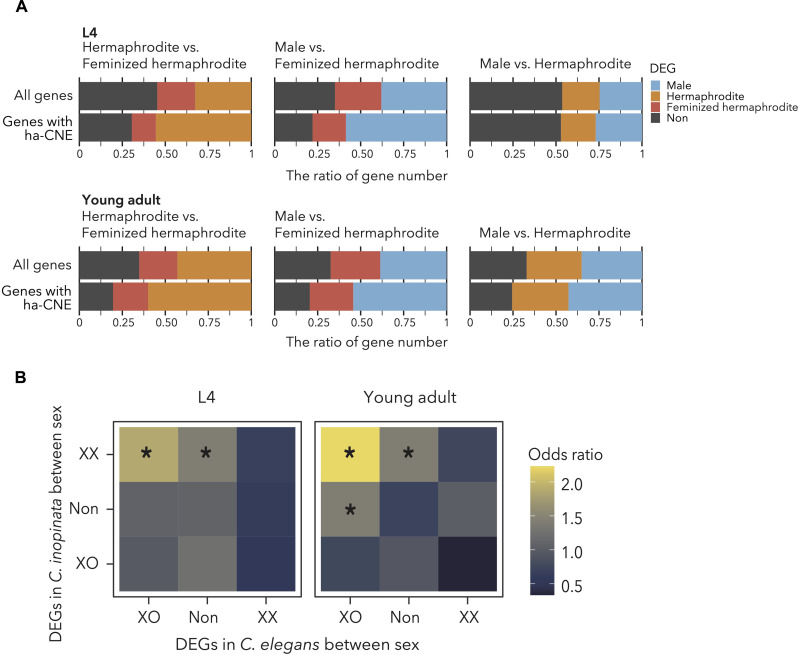
The involvement of ha-CNEs in the neighboring gene expression. (**A**) The relationship between the hermaphrodite-accelerated CNEs and gene expression of *C. elegans*. Each panel shows the percentage of differentially expressed genes (DEGs) between each sex, and color represents the bias of DEGs. No sex bias genes are referred to as “Non.” The upper bars show the percentages of DEGs for all genes in *C. elegans* and the lower bars are only genes neighboring CNEs accelerated in hermaphrodite species. (**B**) The association of ha-CNEs and gene expression transition between *C. elegans* and *C. inopinata*. The genes were classified according to the differentially expressed pattern in two species, and Fisher’s exact test was performed to detect the association of CNE presence/absence neighboring the gene and the proportion of DEGs. The color represents the odds ratio of classified gene sets neighboring ha-CNE. The asterisk represents the significance criteria for Fisher’s exact test (FDR < 0.05).

The most notable difference between dioecious and androdioecious species is the spermatogenesis in hermaphrodites during larval stage 4 (L4) (Fig. 1A). Therefore, to examine the effect of the ha-CNEs on gene regulatory function, the differentially expressed genes (DEGs) were searched between sexes [male versus feminized hermaphrodite (Fem), hermaphrodite versus Fem, and male versus hermaphrodite] in L4 and the young adult stage in *C. elegans* (N2 for males and hermaphrodites, and *hc17* for feminized hermaphrodites) using four replicates of each condition (tables S8 and S10). The principal components analysis (PCA) plots using gene expression levels showed a clear separation of sexes and stages of samples (fig. S4A). The expression profiles of hermaphrodites were intermediate between males and feminized hermaphrodites in L4, and this pattern was consistent with the developmental timing of gametes in *C. elegans*, as expected. Males, hermaphrodites, and feminized hermaphrodites were used to investigate the effect of spermatogenesis- and sperm-related function on gene expression. The genes neighboring ha-CNEs were more frequently highly expressed in both young adult and L4 males versus feminized hermaphrodite mutants (Fig. 3A and table S11), and they were more highly expressed in hermaphrodites when compared between hermaphrodites versus feminized hermaphrodite mutants (Fig. 3A and table S11). These highly expressed genes in male/hermaphrodite patterns are consistent with the presence or absence of sperm, and this difference is likely caused by the gene regulation in sperm and spermatogenesis. Although a weak but statistically significant pattern of highly expressed genes in males was observed in the comparison between males versus hermaphrodites of the young adult stage, no difference was detected in the L4 comparison (Fig. 3A and table S11). These results support that ha-CNEs play a role in regulation of gene expression associated with hermaphrodite development.

### Evolutionary impact on the expression changes associated with the transition to androdioecy

To clarify the relationship between the evolution of hermaphroditism and the impact of ha-CNE on gene expression, we investigated the DEGs between males versus females of *C. inopinata* as dioecious relatives of *C. elegans*. The gene expression profiles of *C. inopinata* were separated by sex and stage as for *C. elegans* (fig. S4B). Plenty of DEGs were detected between sexes in each stage of both species (male versus hermaphrodite in *C. elegans* and male versus female in *C. inopinata*; tables S9 and S10). In the young adult stage, the number of one-to-one orthologs showing different sex-biased expression between the two species was significantly enriched in the genes neighboring ha-CNEs (table S12, one-sided Fisher’s exact test, *P* < 0.01). A similar tendency was observed in L4, although this was not significant (table S12, one-sided Fisher’s exact test, *P* = 0.06528). To further inspect the effect of accelerated evolution on the gene expression changes associated with the evolutionary transition to androdioecy, the association was examined between the ha-CNEs and sex bias of gene expression among species. The one-to-one orthologs between *C. elegans* and *C. inopinata* were classified into several expression categories based on the sex-biased expression in each species (tables S8, S9, and S13). We found that orthologs with no sex bias in *C. elegans* and highly expressed in XX (female) of *C. inopinata* both in L4 and young adults have ha-CNEs more frequently (Fig. 3B and table S13). The orthologs highly expressed in XO (male) *C. elegans* and XX (female) *C. inopinata* also have ha-CNEs more frequently in both L4 and young adults (Fig. 3B and table S13). Assuming dioecy as an ancestral state, these results suggest that ha-CNEs might be involved in diminishing gene expression of XX or enhancing gene expression of XO in hermaphrodite species.

### Genome editing of CNE and monitoring the effect on gene expression pattern

**Fig. 4.**
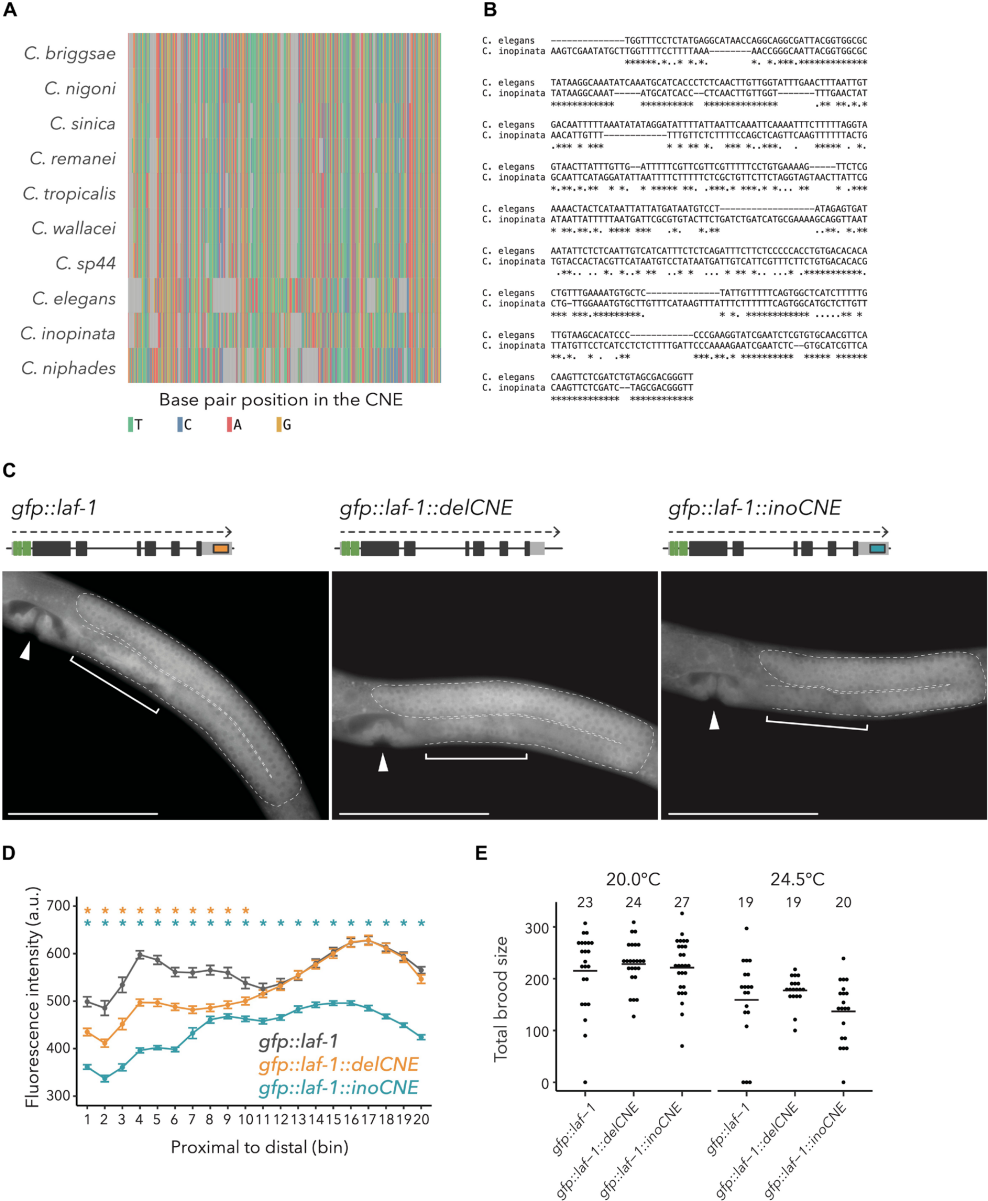
Sequence alignment of the CNE in the *laf-1* 3′ UTR, fluorescent quantification, and fecundity assessment of mutant strains constructed using genome editing. (**A**) Multiple alignment of the CNE among *Caenorhabditis* nematodes. Because the orthologous sequence of *C. latens* was not detected, only 10 species are shown. (**B**) Pairwise alignment of the CNE between *C. elegans* and *C. inopinata*. (**C**) Schematic representations and fluorescent microscope pictures of each *gfp::laf-1* strain. The gonadal region is surrounded by a dashed line. The arrowhead represents the vulva position, and the bracket represents the region of occurring spermatogenesis. All images were taken at 40× magnification. Scale bar, 100 μm. (**D**) Mean values of quantified fluorescent intensity along the gonad of L4 hermaphrodites of each strain. The asterisks at the top of the panel represent the significance of the differences between *gfp::laf-1* versus *gfp::laf-1::delCNE* (yellow) and *gfp::laf-1* versus *gfp::laf-1::delCNE* (blue) by the Wilcoxon rank sum test (FDR < 0.05). (**E**) Total brood size of *gfp::laf-1* mutant animals at 20 and 24.5°C. The numbers at the top in the graph represent the sample size for each strain.

To investigate the involvement of CNEs in the evolution of hermaphroditism, we focused on the CNE in the 3′ UTR of *laf-1*, which was detected as having accelerated evolution in *C. elegans* (Fig. 4, A and B), because this gene is crucial for spermatogenesis in hermaphrodites (Fig. 2). The expression level of LAF-1 in live animals was monitored using the endogenously green fluorescent protein (GFP)::tagged LAF-1 (GFP::LAF-1). Although the fluorescence signals observed were weak through the entire body, they were remarkably intense in the gonads (Fig. 4C, left). To examine the effect of the CNE on LAF-1 expression, the CNE region at the 3**′** UTR was deleted (*gfp::laf-1::delCNE*) and the intensity of fluorescence was measured along the gonad of L4 hermaphrodites. Fluorescence intensity significantly decreased in proximal gonads where spermatogenesis occurs, consistent with the hypothesis that this CNE is involved in the regulation of LAF-1 expression [Fig. 4, C (middle) and D]. Furthermore, when the CNE was replaced with a putative orthologous sequence of the dioecious sister species *C. inopinata* (*gfp::laf-1::inoCNE*), the GFP signal significantly decreased not only in the proximal region but also in the distal region of the gonads [Fig. 4, C (right) and D]. These different effects between deletion and replacement on the expression of LAF-1 suggested that the homologous sequence of these species at the 3**′** UTR has distinct regulatory functions in the gonads. Despite the obvious reduction of fluorescence, no clear difference in total brood sizes was detected between *gfp::laf-1* and either CNE mutant strain (Fig. 4E, *P* > 0.05).

## DISCUSSION

Here, we focused on the association between the evolution of CNEs and the transitions of sexual mode from dioecy to androdioecy that have occurred independently at least three times within *Caenorhabditis* nematodes. The ha-CNEs detected in our analysis, which might include convergently evolved elements, frequently neighbored genes related to sexual phenotype and were involved in the patterns of spermatogenesis-related gene expression in hermaphrodites. Furthermore, comparison of the expression patterns between two species with different sexual modes suggested that the ha-CNEs have an impact on the gene expression changes associated with the evolutionary transition to androdioecy. These results shed light on the contribution of CNEs to the convergent evolution of hermaphroditism and the transition of sexual modes in nematodes.

The CNEs of *Caenorhabditis* nematodes were widespread in the chromosomes, but we detected them more abundantly in chromosome centers. This might be because the distribution of repetitive elements, which cause insertion, deletion, and inversion (*30*, *31*), complicates the genome alignment (Fig. 1, C and E). On the other hand, the small noncoding RNAs (e.g., scRNA, microRNA, and tRNA) and regulatory elements (e.g., enhancers, DNase I hypersensitive sites, and transcription start sites) tend to overlap with CNEs (Fig. 1E). The high sequence conservation of microRNA in *Caenorhabditis* nematodes is consistent with the fact that some families of microRNA have been conserved across animal phylogeny including human (*32*). The CNEs were highly conserved at least among the *C. elegans* wild population (Fig. 1D), suggesting that those regions have functions conserved among this species and are under natural selection. Rapidly evolved CNEs in androdioecious nematodes, i.e., ha-CNEs, frequently overlapped with presumed enhancer regions in the *C. elegans* genome and might be associated with evolutionary changes of neighbor gene expression. The X chromosome has several CNEs, twice as many as found in other chromosomes (Fig. 1C), and this is possibly associated with the peculiarity of the X chromosome in *Caenorhabditis* nematodes, as seen in XO sex determination and germline silencing (*6*, *33*). The X chromosome in *Caenorhabditis* nematodes organizes heterochromatin and exhibits special recombination attributes (*33*), which are probably associated with the high number of CNEs in the X chromosome.

The accelerated evolution of CNEs in each androdioecious nematode showed little overlap among species. However, we showed the possibility that the accelerated evolution of different CNEs neighboring the same genes is associated with the evolution of androdioecy (tables S6 and S7). Although it is possible that the same CNEs had been associated with sexual mode transition convergently, different CNEs neighboring the same gene might cause similar regulatory changes and influence phenotype convergence in each species. Phenotypic association enrichment analysis showed that genes with independently accelerated CNEs in each androdioecious nematode were frequently associated with sexual traits (table S7), implying that those CNEs might be related to the transition to androdioecy of nematodes.

Previous studies have revealed that the sex-determination pathway in *C. briggsae* is at least partly different from that in *C. elegans* (*8*, *28*). Although the sex-determination pathway in *C. tropicalis* has not yet been well studied, genes associated with somatic and germline hermaphroditism in these species may differ from each other. We did not identify sex-determination genes neighboring the CNEs under accelerated evolution in multiple hermaphrodite species. Although only one CNE located in the 3**′** UTR of *laf-1*, which is associated with the onset of spermatogenesis in *C. elegans* L4 hermaphrodites (*8*, *34*), was detected as an ha-CNE, it seems to be subjected to accelerated evolution in *C. elegans*. In contrast, we could not detect the accelerated evolution in the well-studied regulatory region in the *tra-2* 3**′** UTR associated with hermaphrodite spermatogenesis, specifically in *C. elegans* (*35*, *36*). In *C. elegans*, GLD-1 binds to direct repeat elements (DREs) and represses *tra-2* translation; however, other species are presumed not to have acquired this function during evolution (*36*, *37*). The DRE in *tra-2* 3**′** UTR was partially identified as a CNE in our analysis, suggesting that the conserved element acquired a GLD-1 binding region without major sequence changes, i.e., by accelerated evolution, in *C. elegans*. This also implies that such regulatory regions are undetectable by our approach. However, we found that the CNEs rapidly evolved in *C. elegans* and *C. tropicalis* were neighboring to genes in similar pathways of *C. elegans* sex determination (Fig. 2). In both *C. elegans* and *C. tropicalis*, the genes activated during spermatogenesis (*laf-1* and *gld-1*, respectively) have neighboring accelerated CNEs (Fig. 2). We noticed that *fem* genes, the core gene complex of sex determination, were detected in both species (*fem-3* and *fem-2* in *C. elegans* and *C. tropicalis,* respectively). The *fem* gene products physically interact with each other to regulate *tra-1* activity in germ cells (*9*, *38*, *39*). This suggests that independently occurring accelerated evolution in different CNEs around *fem* genes possibly affects the abundance or function of products of the *fem* complex. These findings present the possibility that pathway-level, rather than molecular-level, convergence is associated with the evolution of hermaphroditism for at least two androdioecious nematodes.

Recently, the association between the accelerated evolution of CNEs and convergent phenotypic change was intensely studied (*16*, *19*, *40*–*42*). In our study, we detected accelerated evolution of CNEs in three androdioecious nematodes and revealed that the neighboring genes were associated with the sexual phenotype (table S5). In addition, the neighboring genes showed expression patterns consistent with hermaphrodite-specific spermatogenesis (Fig. 3A). The hermaphrodites of androdioecious nematodes are virtually the same as dioecious females except that they produce sperm in L4 (*5*). Our results indicated that genes neighboring ha-CNEs showed XO-biased expression patterns in the comparison between males and hermaphrodites in young adult, but this pattern was not observed in L4. These expression patterns might be caused by the involvement of ha-CNEs in spermatogenesis because both males and hermaphrodites in L4 are undergoing spermatogenesis. Furthermore, we showed that the ha-CNEs are associated with the gene expression changes between nematodes with different sexual modes (Fig. 3B). The ancestral species of *Caenorhabditis* nematodes seems to be dioecious (*43*, *44*), and if sex-biased expression in the dioecious nematode, *C. inopinata*, is assumed to be the ancestral state, accelerated changes in the ha-CNEs tend to have the effect of enhancing XO-biased or diminishing XX-biased expression in androdioecious nematodes. Previous transcriptomic analysis using feminized mutants showed that androdioecious nematodes have weaker XO-biased expression of genes related to sperm than that in dioecious nematodes, and this might be consistent with the reduction of male function in androdioecious nematodes (*45*). The ha-CNEs are associated with the transition of XX-biased genes to XO-biased genes along with hermaphroditism evolution, and hence ha-CNE is possibly associated with compensation of sperm or male function. Although the detailed impact of these expression patterns is ambiguous, it could have influenced the evolution of hermaphroditism in nematodes.

Our observation using GFP-tagged strains with CNE genome editing showed that the CNE is clearly associated with the regulation of the spermatogenesis-related gene, *laf-1*, in *C. elegans*. The expression level was significantly decreased in the proximal gonads of L4 in the CNE deletion strain. This suggested that the CNE had included a regulatory domain, e.g., repressor site, in the dioecious ancestral lineage, and accelerated evolution of the CNE had resulted in the up-regulation of LAF-1 during spermatogenesis in *C. elegans*. Previous studies have shown that the 3′ UTR is a key regulatory element of gene expression in the germ line (*46*, *47*). Consistent with this previous research, our results suggested that the 3′ UTR of *laf-1* contributes to gene expression during spermatogenesis in hermaphrodites (Fig. 4C). Alternatively, this CNE may influence interactions with ncRNAs. Specifically, it overlaps with the short ncRNA gene (Y71H2AM.28, WBGene00220199). While the function and impact of this ncRNA remains unknown, it is also plausible that this ncRNA is linked to LAF-1 regulation in the gonads. Moreover, the replacement of the CNE into the sister species sequence revealed that the orthologous CNE in dioecious species could not compensate for this reduction of LAF-1 abundance in the gonads, and accelerated evolution of the CNE contributes to gaining an alternative regulatory function (Fig. 4, B and D).

While mutants in CNE neighboring *laf-1* resulted in a change in its expression in the gonadal region undergoing spermatogenesis, those strains showed no differences in total brood size with the GFP-tagged control strain (Fig. 4E). This might be caused by compensation by other redundant genetic factors (*34*) or an insufficient change of the abundance. Previous research also reported that some CNE deletions produced unclear phenotypic changes (*13*, *48*). The decisive criteria or effective screening methods are necessary to predict the effect of each accelerated evolution of CNE. However, the undetectable change in the brood size might be plausible because the evolution of hermaphroditism is presumed to rely on changes in multiple loci during evolution.

Our study supports the hypothesis that the accelerated evolution of CNEs may have played a role in the evolution of gene regulation associated with the sexual mode of *Caenorhabditis* nematodes. The observations made in vivo using CNE mutants strongly suggest that the rapid evolution of CNEs in *C. elegans* plays a crucial role in the regulation of the spermatogenesis-related genes in hermaphrodites. Here, we could not demonstrate the effect of ha-CNE on the brood size; however, it might be plausible that the evolutionary changes in the other several loci are concertedly associated with the transition of sexual modes. Although the function of noncoding elements might be easily compensated by other genetic elements, our study should lead to the development of a system of CNE function in gene regulation that can be examined. These findings contribute to discovering regulatory elements and illuminating the importance of these elements in evolution.

## MATERIALS AND METHODS

### Experimental design

Here, we conducted comparative genomics and transcriptomic analysis to identify ha-CNE and to investigate the effect of ha-CNE on gene regulation. To demonstrate the function of accelerated evolution of CNE, genome editing of a CNE neighboring *laf-1* was conducted.

### Detecting ortholog groups and creating the species tree

To identify orthologs in *Caenorhabditis* nematodes, we used OrthoFinder (*49*) with proteomes of 11 *Caenorhabditis* species: *C. briggsae*, *C. elegans*, *C. latens*, *C. nigoni*, *C. remanei*, *C. sinica*, *C. tropicalis* (obtained from WormBase ParaSite version WBPS15), *C. niphades* (*50*), *C. inopinata* (GCA_003052745.1), *C. sp44*, and *C. wallacei* (http://caenorhabditis.org/). We retained the longest isoforms for genes with multiple isoforms. Single-copy orthologs (4930 genes) were aligned using mafft (*51*), and poorly aligned regions were removed by trimAl with default parameters (*52*). The consensus species tree was estimated from the protein alignment by maximum likelihood with 1000× bootstrapping using IQ tree with automatic substitution model selection (*53*).

### Aligning genome sequences and detecting CNEs

The genome sequences of *Caenorhabditis* nematodes and the species tree inferred from 4930 single-copy orthologs were subjected to multiple whole-genome alignments using Progressive Cactus (*54*). To reduce the bias using *C. elegans* as both the target and reference species, we used *C. niphades* as an outgroup and reference for genome alignment and detection of the CNEs because, like *C. elegans*, *C. niphades* has a high-quality chromosomal-level genome sequence. CNE detection was performed basically in accordance with the methods of a previous study (*16*). The genome alignment with *C. niphades* as a reference was handled by maftools (*55*), and the conserved sequences were detected using PhastCons (with parameters, expected length = 12 and target coverage = 0.3) (*11*). The conserved sequences not overlapping with protein-coding sequences were classified using BEDtools (*56*), and the sequences longer than 10 bp in *C. niphades* genome were defined as CNEs in this study. In the following analysis, we removed the CNE in each species that were aligned redundantly or were more than twice as long as the *C. niphades* sequences. The accelerated evolution of the CNEs was detected by the log-likelihood ratio test implemented in phyloP (*57*). First, to detect accelerated evolution associated with hermaphrodite evolution, all three hermaphrodite branches (*C. elegans*, *C. briggsae*, and *C. tropicalis*) were set as “target” in phyloP at the same run. We also detected accelerated evolution of the CNE in each hermaphrodite branch separately. Enrichment analysis of neighboring genes was performed using the Wormbase Enrichment Suite (*58*).

### Nucleotide conservation in wild populations of *C. elegans*

To investigate whether the CNEs are conserved within species as well as across lineages, we used the SNP dataset published in CeNDR, which is a database of wild isolates of *C. elegans* collected from fields worldwide (*59*). Nucleotide diversity (π) was calculated for CNE and annotated genomic regions based on the category of the general feature format (gff) file of *C. elegans* in WormBase ParaSite (*21*). To reduce the effect of short sequences, we used only sequences >50 bp for calculation.

### RNA sequencing and detection of DEGs between sexes

RNA samples from each sex (hermaphrodite and male for *C. elegans* and male for *C. inopinata*) in L4 and young adult stages were prepared with four replicates of each. *C. elegans* (N2, standard laboratory strain) was cultured at 20°C and fed *Escherichia coli* OP50, and *C. inopinata* (DE3) was cultured at 27°C and fed *E. coli* HT115 for optimal growth (*7*, *60*). For RNA sequencing (RNA-seq), 100 individuals of *C. elegans* and *C. inopinata* were picked and immersed in TRIzol solution with careful treatment to remove *E. coli*. The worms were homogenized and frozen repeatedly several times using BioMasher. The RNA concentration was measured using a Nanodrop 2000 (Thermo Fisher Scientific) and an Agilent Technologies 2100 Bioanalyzer (Agilent Technologies). RNA library construction and sequencing were conducted by outsourcing to Filgen using Novaseq 6000. Raw sequence reads were deposited in the Sequence Read Archive (SRA) under BioProject ID: PRJDB15960. The RNA-seq data of *C. inopinata* females, which were determined under identical conditions to the present study, were retrieved from GenBank (PRJDB14254). To clarify the effect of spermatogenesis and sperm itself in hermaphrodites, we also used the RNA-seq data of the temperature-sensitive feminized mutant *fem-1* (*hc17*) of *C. elegans*. The mRNA abundance of the individual samples was calculated by RNA-Seq by Expectation (RSEM) (*61*) with bowtie2 (*62*) as a mapping program with the default parameter. PCA was performed on transcripts per million (TPM) values using the prcomb function in R, and the global change in gene expression profiles was visualized. The DEGs between sexes were detected using Tag Count Comparison (TCC) (*63*) with edgeR (*64*) with an FDR of less than 0.01. To compare the differences in expression patterns between *C. elegans* and *C. inopinata*, 11,264 one-to-one orthologs estimated by OrthoFinder were used.

### CRISPR-Cas9–mediated genome editing

To construct the strains to monitor *laf-1* expression, a GFP-encoding sequence was inserted at the 5′ end of the coding region of *laf-1* by CRISPR-Cas9–mediated genome editing with a homologous template. The plasmid containing a homologous template (pMN35_laf-1) was constructed by assembling the fragments corresponding to the left homologous recombination (HR) arm (upstream of the start codon, 561 bp), GFP (65C), and the right HR arm (downstream of the start codon, 503 bp) with Bluescript KSII(−). The plasmid that expresses single guide RNA (sgRNA; pTK73_*laf-1*) was constructed by inserting the target sequence for the sgRNA (5′-CAATCGAACAATGGAGGCAG-3′) into the pTK73 vector (*65*). Primers for the homologous template assembly and the guide RNAs are summarized in table S14. The following mixture was injected into the gonads of *C. elegans* N2 adult worms: the homologous repair templates (pMN35_*laf-1*, final concentration = 10 ng/μl), the sgRNA plasmid (pTK73_*laf-1*, 25 ng/μl), the Cas9 expression plasmid pDD162 (Peft-3::Cas9, 50 ng/μl, Addgene no. 47549) (*66*), and the three injection markers pCFJ90 (Pmyo-2::mCherry, Addgene no. 19327), pCFJ104 (Pmyo-3::mCherry, Addgene no. 19328), and pGH8 (Prab-3::mCherry, Addgene no. 19359) (*67*).

For CNE deletion and replacement of the *laf-1* 3′ UTR, the co-CRISPR method with the *dpy-10* gene marker was used (*68*) (fig. S1, A and B). The ribonucleoprotein mix containing recombinant purified *Sp*Cas9 (final concentration = 2.5 μM), sgRNA for *laf-1* (10 μM), and *dpy-10* (2 μM; crRNA:tracRNA = 1:1 mix, IdT) was incubated at 37°C for 10 min. Then, the three injection markers described above, and ssODN for *dpy-10* (10 ng/μl), were added. Following the injection of the mixture, mutants were screened from the progeny of F1 Dpy worms by sequencing the PCR products of the corresponding locus. Sequences for the guide RNA are summarized in table S15.

For CNE replacement, a homologous repair template was constructed by assembling the PCR fragments corresponding to the left HR arm (upstream of *C. elegans* CNE, 945 bp), CNE of *C. inopinata* (468 bp), and the right HR arm (downstream of *C. elegans* CNE, 959 bp) with Bluescript KSII(−) using In-Fusion HD Cloning and then added into the injection mixture (45 ng/μl; fig. S1, A and C). Primers are listed in table S14.

### Fluorescence microscopy

All of the images were taken using an Axioplan 2 imaging microscope (Carl Zeiss, Jena, Germany) with a Plan Apochromat 40×/0.95 objective lens (Zeiss) for worms grown at 20°C. ImageJ/Fiji software (National Institutes of Health, Bethesda, MD) was used to quantify the images of the fluorescent L4 hermaphrodites. For each animal, a line (width = 40 pixels) was drawn starting from the distal tip of the germ line and spanning the entire germ line to the vulva. The fluorescence intensity was measured for each pixel in the line, binned (every 20 pixels), and averaged across each bin in the *z*-slice image in which the distal gonad and vulva are in focus. The differences in mean fluorescence intensities were tested by the Wilcoxon rank sum test using a statistical package in R.

### Brood size

For each strain, L4 hermaphrodites were placed on 35-mm nematode growth medium agar plates seeded with *E. coli* OP50. Each animal was moved to a fresh plate twice per day until the completion of the experiment. The number of eggs and larvae on the plate was counted after removing the animals. The number of progeny was calculated as the total number of eggs and larvae produced during the animal’s fertile period. Animals were grown and scored at optimal and high temperature, 20° and 24.5°C, respectively, to test whether mutants are temperature sensitive or not. The differences in the number of progeny between the *gfp::laf-1* strain and each CNE mutant were tested by the Wilcoxon rank sum test.

### Statistical analysis

All statistical analyses were performed using R version 4.2.1.
