# Supplementary material for: Evolutionary changes of noncoding elements associated with transition of sexual mode in Caenorhabditis nematodes
Source: Sci Adv. 2024 Sep 13;10(37):eadn9913. doi: 10.1126/sciadv.adn9913 (PMC11397494; doi:10.1126/sciadv.adn9913)
Supplement: Supplementary file 1 — Figs. S1 to S4 Legend for data S1 [file sciadv.adn9913_sm.pdf]

Science Advances
AAAS

Supplementary Materials for

Evolutionary changes of noncoding elements associated with transition of sexual mode in *Caenorhabditis* nematodes

Katsunori Tamagawa *et al.*

Corresponding author: Katsunori Tamagawa, tamagawa.katsunori@gmail.com; Asako Sugimoto, asugimoto@tohoku.ac.jp; Takashi Makino, tamakino@tohoku.ac.jp

Sci. Adv. **10**, eadn9913 (2024)
DOI: 10.1126/sciadv.adn9913

The PDF file includes:

Figs. S1 to S4
Legend for data S1

Other Supplementary Material for this manuscript includes the following:

Data S1

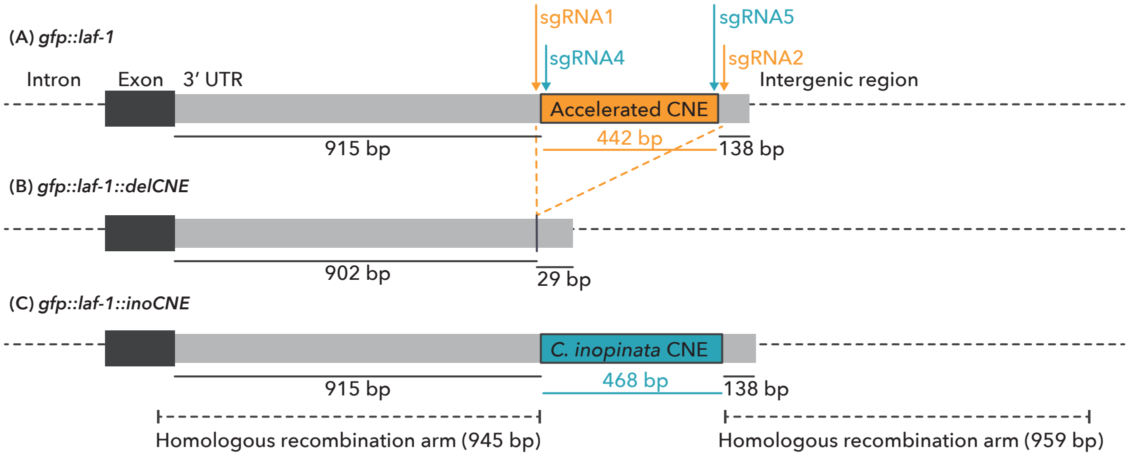

Fig. S1.

Schematic representing the 3′UTR of *gfp::laf-1* (A), *gfp::laf-1::delCNE* (B), and *gfp::laf-1::inoCNE* (C). The black rectangle is the last exon and grey is 3′UTR of *laf-1*.

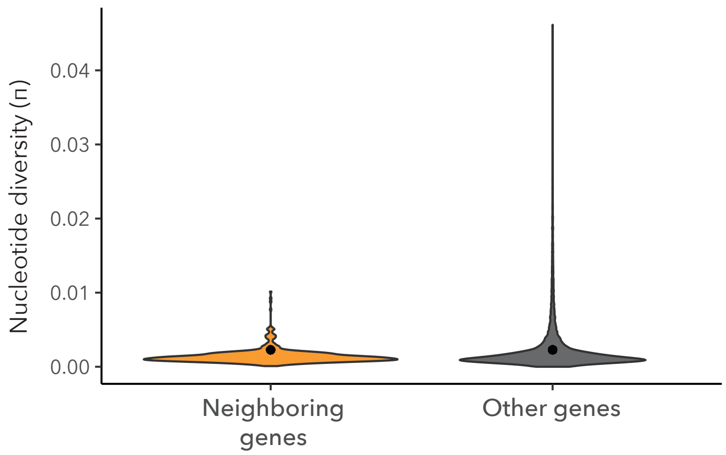

Fig. S2.

Figure S2. The nucleotide diversity (π) of genes neighboring to ha-CNE and other genes.

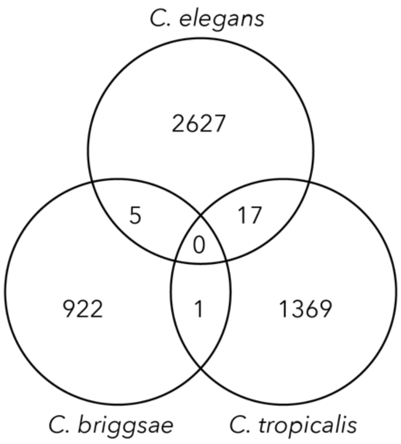

Fig. S3.
Venn diagram of the number of the CNEs detected as accelerated evolved by phyloP in each hermaphrodite species.

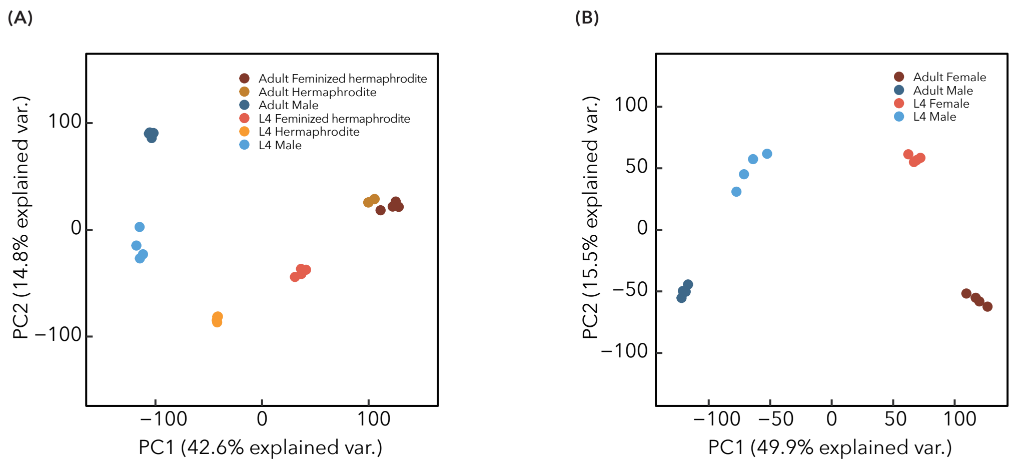

Fig. S4.

The principal component analysis (PCA) plot of TPM values of gene expression in *C. elegans* (A) and *C. inopinata* (B). Each point is a replicate of the sample and the colors represent the sexes and stages of samples.

Data S1. (separate file)
Supplementary table S1 to S15.

Table S1.
The number of CNEs in each species.

Table S2.
The number and the base-pair percentage of CNEs in the *C. elegans* genome.

Table S3.
Overlap of ha-CNE and regulatory elements in *C. elegans* genome (Fisher's-exact test).

Table S4.
Gene id list of *C. elegans* neighbor to ha-CNEs detected by PhyloP (FDR < 0.05)

Table S5.
Result of gene set enrichment analysis using genes neighbor to ha-CNEs (Q-value < 0.05).

Table S6.
Gene id list neighboring to CNEs detected in three species independently.

Table S7.
Result of gene set enrichment analysis using genes neighbor to CNEs accelerated in three androdioecious species independently (Q-value < 0.05)

Table S8.
List of differentially expressed genes in *C. elegans* (FDR < 0.01).

Table S9.
List of differentially expressed genes in *C. inopinata* (FDR < 0.01).

Table S10.
The number of differentially expressed genes between sexes (Q-value < 0.01)

Table S11.
Association of ha-CNEs and sex-biased genes in *C. elegans* (one-sided Fisher's-exact test).

Table S12.
Association between ha-CNEs and gene showing different sex-biased patterns between *C. elegans* and *C. inopinata* (one-sided Fisher's-exact test).

Table S13.
Association between ha-CNEs and transition of sex-biased expression pattern between *C. elegans* and *C. inopinata* (Fisher's-exact test).

Table S14.

Primer list for sgRNA and homologous repair templates

Table S15.
Sequence list for sgRNA
